# Effectiveness of Web-Based Mindfulness-Based Interventions for Patients With Cancer: Systematic Review and Meta-Analyses

**DOI:** 10.2196/47704

**Published:** 2024-06-25

**Authors:** Ting Wang, Chulei Tang, Xiaoman Jiang, Yinning Guo, Shuqin Zhu, Qin Xu

**Affiliations:** 1 School of Nursing, Nanjing Medical University Nanjing China

**Keywords:** cancer, mindfulness-based interventions, mental health, randomized controlled trial, systematic review, meta-analysis, mindfulness, web-based intervention, oncology, delivery mode, efficacy, quality of life, program, adherence, mobile phone

## Abstract

**Background:**

Cancer has emerged as a considerable global health concern, contributing substantially to both morbidity and mortality. Recognizing the urgent need to enhance the overall well-being and quality of life (QOL) of cancer patients, a growing number of researchers have started using online mindfulness-based interventions (MBIs) in oncology. However, the effectiveness and optimal implementation methods of these interventions remain unknown.

**Objective:**

This study evaluates the effectiveness of online MBIs, encompassing both app- and website-based MBIs, for patients with cancer and provides insights into the potential implementation and sustainability of these interventions in real-world settings.

**Methods:**

Searches were conducted across 8 electronic databases, including the Cochrane Library, Web of Science, PubMed, Embase, SinoMed, CINAHL Complete, Scopus, and PsycINFO, until December 30, 2022. Randomized controlled trials involving cancer patients aged ≥18 years and using app- and website-based MBIs compared to standard care were included. Nonrandomized studies, interventions targeting health professionals or caregivers, and studies lacking sufficient data were excluded. Two independent authors screened articles, extracted data using standardized forms, and assessed the risk of bias in the studies using the Cochrane Bias Risk Assessment Tool. Meta-analyses were performed using Review Manager (version 5.4; The Cochrane Collaboration) and the *meta* package in R (R Foundation for Statistical Computing). Standardized mean differences (SMDs) were used to determine the effects of interventions. The Reach, Effectiveness, Adoption, Implementation, and Maintenance framework was used to assess the potential implementation and sustainability of these interventions in real-world settings.

**Results:**

Among 4349 articles screened, 15 (0.34%) were included. The total population comprised 1613 participants, of which 870 (53.9%) were in the experimental conditions and 743 (46.1%) were in the control conditions. The results of the meta-analysis showed that compared with the control group, the QOL (SMD 0.37, 95% CI 0.18-0.57; *P*<.001), sleep (SMD −0.36, 95% CI −0.71 to −0.01; *P*=.04), anxiety (SMD −0.48, 95% CI −0.75 to −0.20; *P*<.001), depression (SMD −0.36, 95% CI −0.61 to −0.11; *P*=.005), distress (SMD −0.50, 95% CI −0.75 to −0.26; *P*<.001), and perceived stress (SMD −0.89, 95% CI −1.33 to −0.45; *P*=.003) of the app- and website-based MBIs group in patients with cancer was significantly alleviated after the intervention. However, no significant differences were found in the fear of cancer recurrence (SMD −0.30, 95% CI −1.04 to 0.44; *P*=.39) and posttraumatic growth (SMD 0.08, 95% CI −0.26 to 0.42; *P*=.66). Most interventions were multicomponent, website-based health self-management programs, widely used by international and multilingual patients with cancer.

**Conclusions:**

App- and website-based MBIs show promise for improving mental health and QOL outcomes in patients with cancer, and further research is needed to optimize and customize these interventions for individual physical and mental symptoms.

**Trial Registration:**

PROSPERO CRD42022382219; https://www.crd.york.ac.uk/prospero/display_record.php?RecordID=382219

## Introduction

### Background

The 2020 Global Cancer Statistics Report estimates that there are 19.3 million new cases of cancer worldwide and approximately 10 million cancer-related deaths [1]. The leading cause of disease and mortality among humans today is cancer [2,3]. The physical symptoms of patients with cancer have been alleviated because of the continuous advancement of medical technology, but the psychological problems of patients with cancer have not been adequately treated. The process of treating cancer is typically complex, with many patients experiencing negative side effects of cancer treatments, such as chemotherapy and radiation therapy, that may impact their mental health, quality of life (QOL), and sleep quality. Targeted interventions to address these cancer-related symptoms can reduce the psychological burden of cancer treatment and diagnosis, which is critical to improving patients’ QOL and promoting their health [4]. With an increasing number of patients with cancer and a desire for physical and mental health, cancer care research is focusing on identifying the psychological problems of patients with cancer and developing and implementing patient-centered psychological care plans [5,6]. Rehabilitation for patients with cancer increasingly uses mental health as a therapeutic strategy; however, effective psychological intervention strategies are still urgently needed to satisfy the demands of patients with cancer [7].

Mindfulness-based interventions (MBIs) have emerged as promising intervention techniques for patients with cancer. Mindfulness can be defined as the ability to observe thoughts, bodily sensations, or feelings in the present moment with an open and accepting orientation toward one’s experiences [8]. MBIs, which incorporate mindfulness practices into various therapies in mental health care, have been found to increase psychological flexibility and alleviate intense emotional states. MBIs can include additional mental training, such as mindfulness-based stress reduction (MBSR) [9], and acceptance and commitment therapy [10], which addresses psychological issues by increasing psychological flexibility [11]. Cognitive-behavioral therapy has been combined with MBSR, resulting in mindfulness-based cognitive therapy (MBCT) for preventing depression relapses [12]. Mindfulness-based cancer recovery (MBCR), an adaptation of MBSR, comprises contents tailored for patients with cancer [13]. Through facilitating awareness and nonjudgmental acceptance of moment-to-moment experiences, these MBIs are presumed to alleviate intense emotional states. Mindfulness interventions have been shown to improve the psychological status of patients with cancer [14,15].

The rapid development of information technologies has led to the delivery of MBIs via the internet, which is more practical than face-to-face interaction and can overcome time and geographic barriers, and it has been established that online MBIs are more suitable for people with psychological and physical symptoms [16]. Implementing psychological interventions through online or remote health can be a potential cost benefit for current referral pathways and treatment models [17]. online MBIs can be used as the adjunctive therapy in patients with cancer to manage cancer-related symptoms [18].

Despite the increasing popularity of online mindfulness-based therapies for patients with cancer and the growing number of randomized controlled trials (RCTs) examining such programs, there has not been a systematic review of these studies and their descriptions of the interventions regarding their characteristics (eg, delivery mode and approach). To date, only 2 systematic reviews addressing the impact of online interventions on health outcomes in patients with cancer have been published. However, these reviews have notable limitations. The first review [19] only searched 4 databases, potentially leading to publication bias and compromising the reliability of the findings. Furthermore, this systematic review did not conduct sensitivity, subgroup, or meta-analyses. The second review [20] evaluated the validity of online MBIs on only 4 health outcomes: anxiety, depression, QOL, and mindfulness. However, the restricted quantity of RCTs and papers within each subgroup analysis poses a challenge in reaching definitive conclusions. In addition, the external validity (eg, generalizability or applicability) based on the RE-AIM (Reach, Effectiveness, Adoption, Implementation, and Maintenance) framework has not been examined in online MBIs for patients with cancer. Thus, attempts to synthesize the literature on the impact of online MBIs on the health of patients with cancer are limited, and there is a lack of analysis of the barriers and facilitators to the development of current online MBIs.

### Objectives

This systematic review aims to synthesize the effectiveness of online MBIs, encompassing both app- and website-based MBIs, for patients with cancer, comprehensively assessing a wide range of outcomes, including psychological, physiological, and QOL aspects. We conducted a comprehensive search to evaluate the validity of app- and website-based MBIs on psychological outcomes in patients with cancer, using high-quality RCTs to assess many health outcomes before and after treatment. Moreover, this study aims to provide an overview of the outcomes related to the interventions, including their effectiveness and potential for implementation and sustainability in real-world settings. We used the RE-AIM framework [21] to evaluate the potential for implementation and sustainability of these interventions in real-world settings. Using this framework, we can provide a comprehensive evaluation of an intervention’s potential impact and identify common traits of effective interventions. Overall, this study fills gaps in the literature by comprehensively evaluating the effectiveness and potential for implementation and sustainability of app- and website-based MBIs for patients with cancer.

## Methods

### Search Strategy

The protocol of this review was registered in PROSPERO (CRD42022382219) and written following the PRISMA (Preferred Reporting Items for Systematic Reviews and Meta-Analyses) reporting guideline. The methods outlined in the protocol were strictly adhered to throughout the experimental procedures. The databases were searched until December 30, 2022. To identify relevant studies for inclusion in our systematic reviews, we developed comprehensive search strategies and used 8 databases: Cochrane Library, Web of Science, PubMed, Embase, SinoMed, CINAHL Complete, Scopus, and PsycINFO. The literature search language was limited to Chinese and English. The search strategies used a combination of subject headings (eg, Medical Subject Headings in PubMed) and keywords for the following 5 concepts: mindfulness, carcinoma, intervention, telemedicine, and randomly. Multimedia Appendix 1 shows detailed database search strategies. Reference lists of included studies and relevant systematic reviews were also manually searched for additional relevant studies. Search results were captured using citation management software, and duplicates were removed.

### Inclusion and Exclusion Criteria

Because of the explorative nature of this meta-analysis, we opted for rather broad inclusion criteria. The inclusion criteria were as follows: (1) studies that included patients with cancer (aged ≥18 years) with any cancer type and stage, including those receiving anticancer treatment, those in remission, those considered cured, and those in the terminal phases of the disease; (2) studies that used MBIs (including MBSR, MBCT, and MBCR) and administered the MBI via the internet (including websites, web conferencing, web-based games, and web-based video) or a smartphone app; (3) studies in which eligible controls were required to receive standard care or usual care; (4) studies were eligible if a mental health outcome (eg, fear of cancer recurrence [FCR] as measured with the Fear of Cancer Recurrence Inventory [FCRI] and posttraumatic growth [PTG] as measured with the Posttraumatic Growth Inventory), anxiety, depression, distress, stress, and sleep) or QOL was assessed; and (5) the RCT was published in English or Chinese.

Exclusion criteria were (1) other types of studies (eg, observational, review, protocol, and case report); (2) studies of health professionals, caregivers, or mixed populations in which outcomes for survivors of cancer could not be extracted; and (3) insufficient information to calculate an effect size or determine eligibility.

### Screening and Data Extraction

Two reviewers independently screened all titles and abstracts; then, they independently screened full-text articles, and conflicts were resolved by consensus. Data were independently extracted by 2 reviewers using a data extraction form adapted from the Cochrane Handbook [22] and reported using PRISMA guidelines [23]. We extracted data from included trials using standardized data extraction forms. Study-level variables included the year of publication, country of study, age of participants, cancer diagnosis, delivery mode, reminder, cancer-adapted MBIs, primary and secondary outcomes, intervention and follow-up durations, intervention and control group details, outcome measurement metrics, and outcomes scores up to postintervention. Any discrepancy or uncertainties were resolved through regular meetings and discussion among the research team.

### Risk-of-Bias Assessment

The risk of bias was independently assessed by 2 reviewers using the Cochrane Risk-of-Bias tool, with differences reconciled through discussion [24]. A total of 6 domains encompassed random sequence generation, allocation concealment, blinding of participants and personnel, blinding of outcome assessment, incomplete outcome data, and selective outcome reporting. Each domain was judged as low, high, or unclear risk. Discrepancies in assessments between the 2 reviewers were resolved by consensus or by a third reviewer as required.

### Meta-Analytic Method

This study conducted a meta-analysis using Review Manager (version 5.4; The Cochrane Collaboration) and the *meta* package in R (R Foundation for Statistical Computing). The primary and secondary outcome mean and SD values at postintervention follow-up for intervention and control groups were converted to standardized mean difference (SMD), using Hedges *g*. The value of SMD <0.5 would be interpreted as small, SMD ≥0.5 as medium, and SMD ≥0.8 as large effect size [25]. Authors of studies with missing data were contacted through email. However, if no data were provided, a narrative synthesis would be conducted. The *I*^2^ statistic was used to estimate the percentage of heterogeneity across the primary studies not attributable to random sample error alone. A value of 0% indicated no heterogeneity, and values of 25%, 50%, and 75% reflected low, moderate, and high degrees of heterogeneity, respectively [26]. Acknowledging differences across studies because of the varied population, length of intervention, and length of follow-up, meta-analyses were performed fitting random effects models [27]. In addition, subgroup analyses were conducted to examine effect sizes across different subgroups; the specific moderating variables included technology, sex, intervention type, intervention duration, study quality, and scale.

### RE-AIM Framework

The RE-AIM framework is a valuable tool for evaluating interventions in health care [28]. Its 5 dimensions assess an intervention’s potential for large-scale adoption, implementation, and sustainability, providing a comprehensive evaluation of its real-world efficacy and viability [29]. Reach refers to the extent of successfully targeting and engaging the intended audience, evaluated using the percentage of eligible patients enrolled in the study (n enrolled/n eligible). Efficacy measures the effect on outcomes such as mental health and QOL. Effect sizes (95% CIs) for the primary outcome were used to assess efficacy. Adoption measures the extent to which organizations or health care providers are willing and able to offer the intervention to their patients or clients, and barriers to adoption are evaluated by who recruited participants and where the intervention was offered. Implementation evaluates how effectively the intervention is delivered and received by patients, including factors such as adherence and fidelity, and is evaluated by measures such as adherence to the intervention, percentage of dropouts of the most complex intervention (n postintervention follow-up/n baseline×100), intervention cost, and author-reported plans to upscale or implement. Maintenance measures the extent to which the intervention can be sustained over time and integrated into routine care, and it is evaluated by the duration of results and the author-reported availability of the intervention [30].

## Results

### Description of Studies

**Figure 1:**
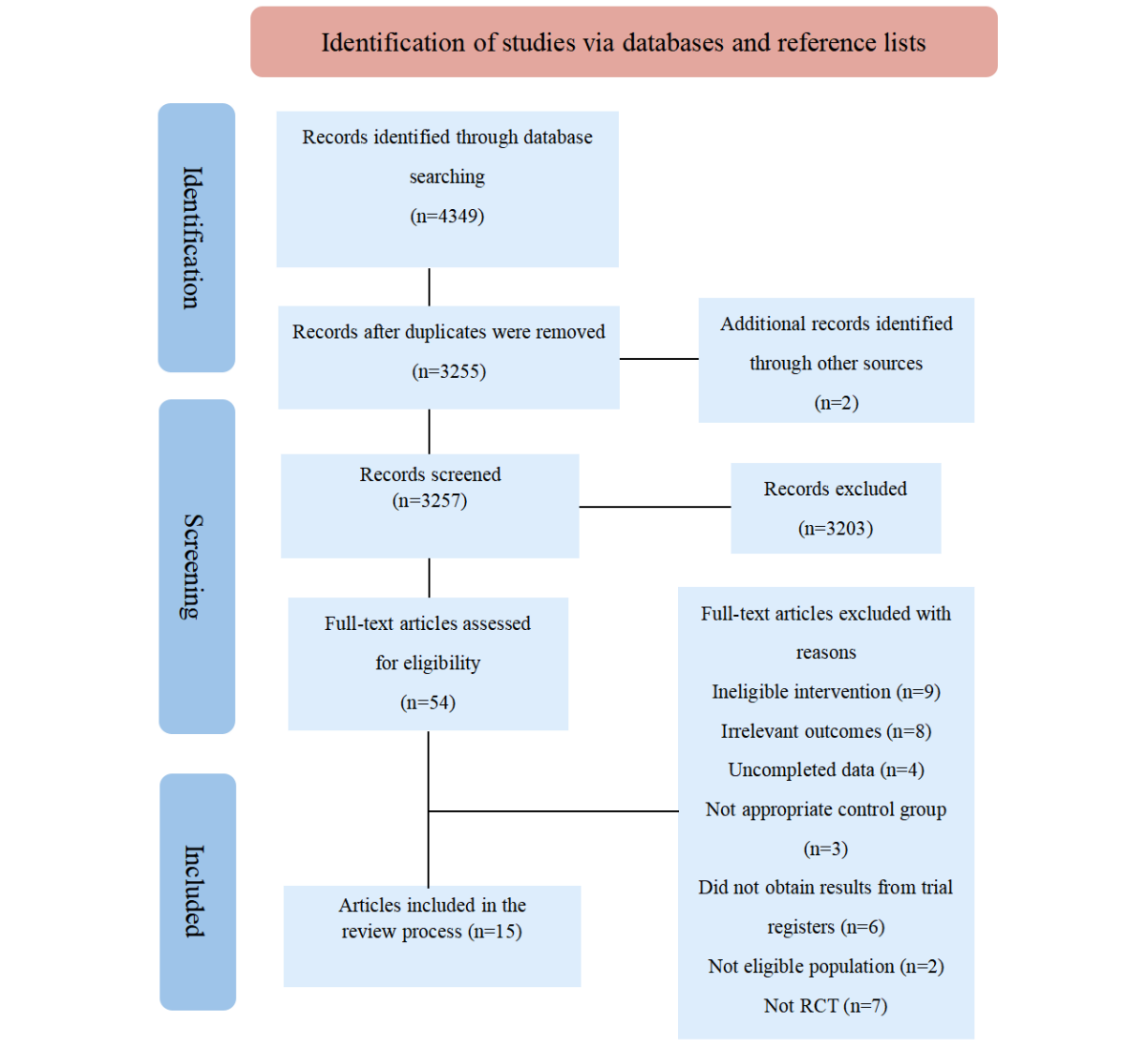
Flow diagram of trial identification and selection. RCT: randomized controlled trial.

The systematic search revealed 4349 original articles, of which 54 (0.12%) were assessed at full-text level, and 15 (0.03%) studies were included in the final synthesis. Figure 1 displays the study flowchart of the search results and Table 1 presents the characteristics extracted from the included literature in the study. The total population comprised 1613 participants, of which 870 (53.94%) and 743 (46.06%) were in the experimental and control conditions, respectively. In most (13/15, 87%) studies, the majority of participants were women. Participants were aged from 41.84 to 66.45 years. Four studies were based on MBCR, 3 on MBCT, 2 on MBSR, and 6 on mindfulness-based programs. The 6 studies included interventions that were indeed rooted in mindfulness practices; however, they did not strictly adhere to the conventional frameworks of MBCT, MBCR, or MBSR. Instead, they used a variety of mindfulness-based approaches tailored to their respective study populations.

Furthermore, these studies did not specify the exact intervention methods used but were categorized as *mindfulness-based programs*. Because of the unique nature of these interventions, we cannot determine whether they belong to MBCT, MBCR, or MBSR interventions; we have categorized them as *mindfulness-based programs*, encompassing diverse methodologies beyond the traditional MBCT, MBCR, or MBSR frameworks. Trials used usual care (8 trials) and waitlists (7 trials) equally as comparators. Six studies had participants with breast cancer, 7 with mixed cancer types, and 2 with other cancer types. Five studies were conducted in China; 5 in the United States; and 1 each in the Netherlands, Denmark, Iran, Australia, and Canada.

**Table 1:** Characteristics of the included studies.

Study; country	Cancer type; age (y), mean (SD); gender (female; %)	Intervention (n); delivery mode	Reminders	Intervention duration, number of sessions; intervention dose	Cancer adapted	Technology	Control group (n)	Measurements	Outcomes: measure instrument
Chang et al [31], 2022; China	Breast; 49.6 (12.0); 100	MBSR^a^ (41); web-based software and digital interactive whiteboard	—^b^	6 wk, 6 sessions; 2 h/wk	—	Website	Waitlist (26)	Pre and post	Depression, anxiety, and stress: DASS-21^c^
Compen et al [32], 2018; Netherlands	Mixed; 51.7 (10.7); 85	MBCT^d^ (90); email, meditation audio file, and written feedback	—	8 wk; 40-45 min/d twice daily	—	Website	Usual care (78)	Pre and post	Distress: HADS^e^, FCR^f^: FCRI^g^, and QOL^h^: SF-12^i^
Kubo et al [33], 2019; US	Mixed; 58.2 (14.4); 68	MBP^j^ (54); audio instruction, lecture videos, and foundation course	Study staff made phone calls if an intervention participant completed <3	8 wk, 2 h/wk	Specifically for individuals affected by cancer	App	Usual care (43)	Pre and post	Distress: NCCN^k^, anxiety and depression: HADS, PTG^l^: PTGI^m^, sleep: PROMIS^n^, and QOL:FACT-G^o^
Kubo et al [34], 2018; US	Mixed; 66.5 (9.7); 69	MBP (52); online classroom and manual	The app can send reminders using push notifications; study staff made phone calls if an intervention participant completed <3	6 wk; 2 h/wk	Cancer pack, which was designed specifically for individuals affected by cancer	App	Usual care (51)	Pre and post	QOL: FACIT−Pal^p^, distress: NCCN, and anxiety and depression: HADS
Liu et al [35], 2022; China	HCC^q^; 55.7 (8); 22	MBCT (61); WeChat audio and online platforms	Every day (texting)	6 wk, 20 min/d for 5 d/wk	Adoption of the main issues and needs of patients with HCC	App	Waitlist (61)	Pre, post, 1 mo FU^r^, 3 mo FU	Distress: HADS, sleep: PSQI^s^, QOL: FACT-hep^t^, and stress: PSS^u^
Messer et al [36], 2020; US	Mixed; 51 (10.6); 76	MBSR (11); guided meditation audio clips and brief textual lessons	—	6 wk; mean duration of 12 min/session	—	Website	Usual care (10)	Pre and post	Distress: HADS, QOL: POMS-SF^v^, and sleep: PSQI
Nissen et al [37], 2018; Denmark	Breast and prostate; 55.9 (12.1); 91	MBCT (104); website written material, audio exercises, writing tasks, and videos	—	10 wk, 10 sessions; 2 h/wk for 45 min/d	Program adjustments to meet the needs of survivors of cancer	Website	Waitlist (46)	Pre, post, and 6 mo FU	Anxiety: STAI‐Y^w^, depression: BDI‐II^x^, stress: PSS‐10, and sleep: ISI^y^
Peng et al [38], 2022; China	Breast; 41.8 (2.9); 100	MBP (30); website meeting 5P^z^ medicine approach	—	6 wk, 6 sessions; 1.5 h/wk	On the basis of specific considerations for survivors of cancer	App	Usual care (30)	Pre, post, and 1 mo FU	FCR: FCRI-SF^aa^ and QOL: Eortc-Qlq-C30^a^^b^
Rosen [39], 2017; US	Breast; 53 (10.3); 100	MBP (48); app-based courses include audio and video	General weekly check-in emails	9 wk	—	App	Waitlist (47)	Pre, wk 5, wk 9, and wk 4 FU	QOL: FACT-B^a^^c^
Rosen et al [40], 2018; US	Breast; 51.6 (10.3); 100	MBP (57); app-based audio and animated video	Weekly check-in email	9 wk	—	App	Waitlist (55)	Pre, post, and 1 mo FU	QOL: FACT‐B
Russell et al [41], 2019; Australia	Melanoma; 53.4 (13.1); 54	MBP (46); embedded short videos, PDF transcript of the videos, and MP3 audio email	Automatically generated email reminders twice daily	6 wk	Survey to understand the knowledge of meditation among people with melanoma	Website	Waitlist (23)	Pre and post	FCR: FCRI and stress: PSS-10
Shen et al [42], 2021; China	Breast; 47.4 (7.5); 100	MBCR^a^^d^ (37); online course, WeChat group, audio-video materials, and pictures	Every day (texting)	8 wk, 8 sessions; 15 min/d for 6 d/wk	Combine rich experience in rehabilitation psychotherapy of breast cancer	App	Usual care (40)	Pre and post	Stress: CPSS^a^^e^ and anxiety: SAS^a^^f^
Wang [43], 2022; China	Breast; 46.8 (7.9); 100	MBCR (51); web-based courses and intervention materials	—	4 wk, 4 sessions; 1.5 h/wk and 30 min daily	On the basis of the problems in the pilot study and participant feedback, adjusted internet-delivered MBCR program	Website	Usual care (52)	Pre and post	QOL: FACT-B
Yousefi et al [44], 2022; Iran	Colorectal and stomach; 54.9 (6.6); 42	MBCR (25); web-based session	An alert reminder message was sent 2 h before each session	9 wk, 9 sessions; 90 min/wk	Cancer-specific MBSR program was used in the study	Website	Usual care (25)	Pre, post, and 2 mo FU	Stress: DASS‐21 and sleep: ISI
Zernicke et al [45], 2014; Canada	Mixed; 58 (10.7); 72	MBCR (30); web-based classroom, guided meditation recordings, and videos	—	8 wk, 8 sessions; 45 min/d	Cancer-adapted MBSR	Website	Waitlist (32)	Pre and post	Depression and anxiety: POMS^a^^g^, stress: CSOSI^a^^h^, PTG: PTGI

^a^MBSR: mindfulness-based stress reduction.

^b^Not applicable.

^c^DASS-21: Depression, Anxiety, and Stress Scale-21.

^d^MBCT: mindfulness-based cognitive therapy.

^e^HADS: Hospital Anxiety and Depression Scale.

^f^FCR: fear of cancer recurrence.

^g^FCRI: Fear of Cancer Recurrence Inventory.

^h^QOL: quality of life.

^i^SF-12: 12-item Short-Form health survey.

^j^MBP: mindfulness-based program.

^k^NCCN: National Comprehensive Cancer Network Distress Thermometer.

^l^PTG: posttraumatic growth.

^m^PTGI: 21-item Posttraumatic Growth Inventory.

^n^PROMIS: 8-item PROMIS Sleep Disturbance scale.

^o^FACT-G: 27-item Functional Assessment of Cancer Therapy General Scale.

^p^FACIT‐Pal: 46-item Functional Assessment of Chronic Illness Therapy—Palliative Care.

^q^HCC: hepatocellular carcinoma.

^r^FU: follow-up.

^s^PSQI: Pittsburgh Sleep Quality Index.

^t^FACT-Hep: Functional Assessment of Cancer Therapy-Hepatobiliary Carcinoma.

^u^PSS: Perceived Stress Scale.

^v^POMS-SF: Profile of Mood States-Short Form.

^w^STAI‐Y: State-Trait Anxiety Inventory Y-Form.

^x^BDI‐II: Beck Depression Inventory.

^y^ISI: Insomnia Severity Index.

^z^5P: The specific name of an application designed to promote mind and brain health and cultivate happiness.

^aa^FCRI-SF: Fear of Cancer Recurrence Inventory-Short Form.

^ab^Eortc-Qlq-C30: European Organization for Research and Treatment of Cancer questionnaire.

^ac^FACT-B: Functional Assessment of Cancer Therapy-Breast version 4.

^ad^MBCR: Mindfulness-based cancer recovery.

^ae^CPSS: Chinese version of the Perceived Stress Scale.

^af^SAS: Self-Rating Anxiety Scale.

^ag^POMS: Profile of Mood States.

^ah^CSOSI: Calgary Symptoms of Stress Inventory.

### Risk of Bias

**Figure 2:**
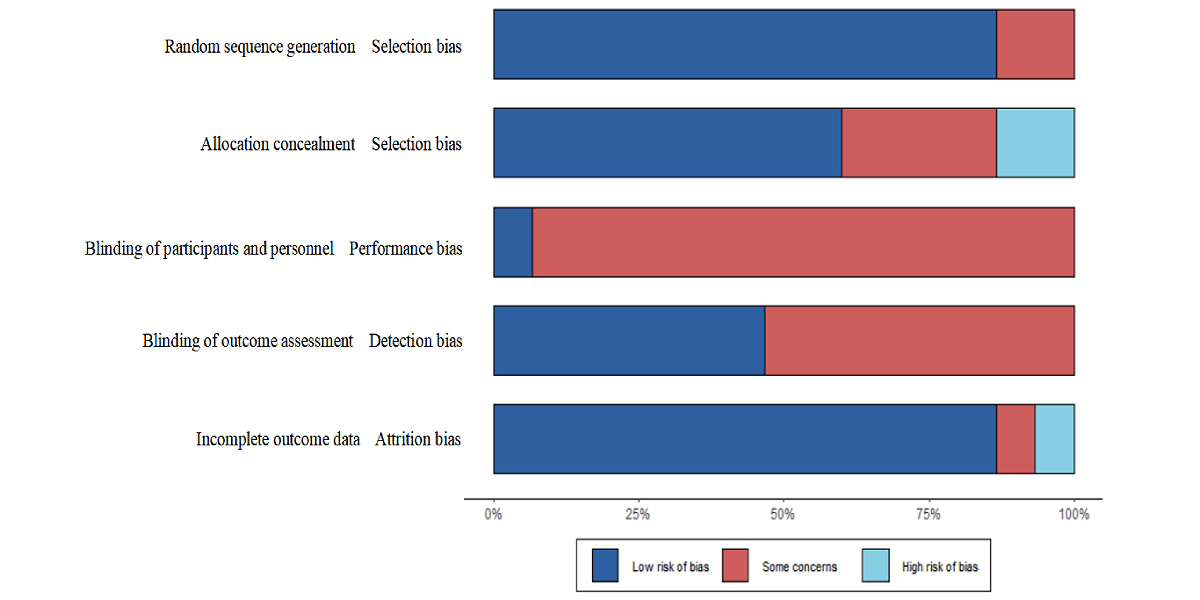
Cochrane risk-of-bias scores (percentage of low, unclear, and high risk) across bias domains (selective reporting, incomplete outcome data, blinding, allocation concealment, and random sequence generation) for the 15 included web-based mindfulness-based intervention studies for patients with cancer.

The risk-of-bias assessment is presented in Multimedia Appendix 2 [31-45]. Most studies (9/15, 60%) adequately generated and concealed allocation (Figure 2). In most studies (14/15, 93%), patient blinding was not possible because of the nature of online MBIs and was not considered to increase the risk of bias. However, of the 15 studies, 8 (53%) [31,33,37-40,42,44] presented insufficient information regarding researcher and outcome assessor blinding, whereas 7 (47%) reported blinding researchers [32,34-36,41,43,45] (low risk). A total of 14 studies reported complete outcome data (low risk), and 1 study had insufficient detail [44] (unclear risk). In 1 study [40], attrition was high and comparisons or reasons for attrition were not provided. Finally, 66% (10/15) of the studies did not reference a protocol or trial registration (unclear risk).

### Meta-Analysis

#### Effects on QOL

**Figure 3:**
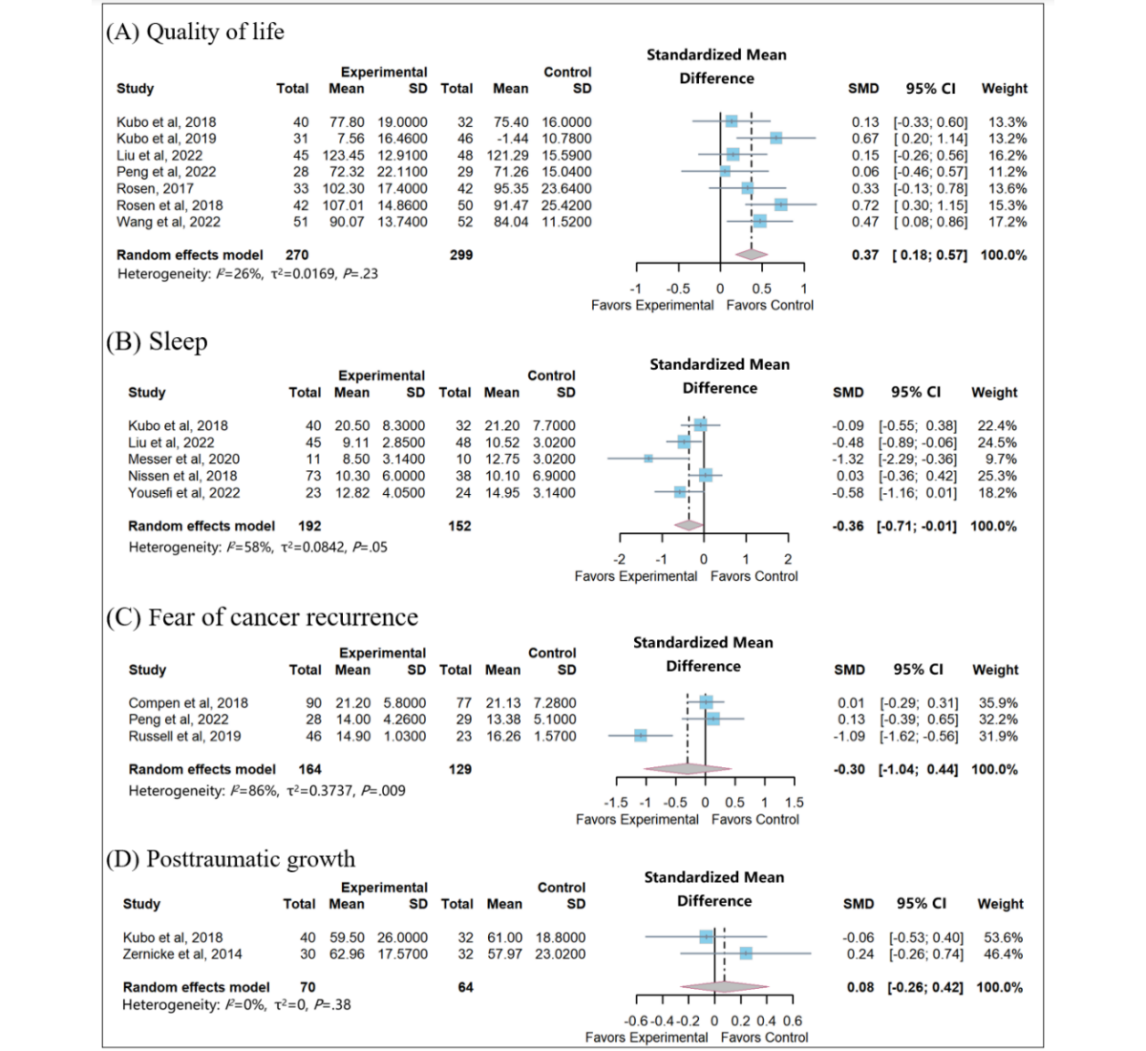
Meta-analysis on (A) quality of life, (B) sleep, (C) fear of cancer recurrence, and (D) posttraumatic growth. SMD: standardized mean difference.

A total of 8 studies reported the effects of app- and website-based MBIs on QOL among patients with cancer. To measure QOL in patients with cancer, 4 health-related QOL measures were used, including the Functional Assessment of Chronic Illness Therapy [34], the Functional Assessment of Cancer Therapy [33,35,39,40,43], the Short-Form 12 [32], and the European Organization for Research and Treatment of Cancer questionnaire [38], all of which have been validated in this patient population. Higher scores reflected a higher QOL. Because the physical and psychological components of the scale were measured separately and it was not possible to determine the overall change in the QOL, the data from 1 study [32] were not summarized. A total of 7 studies including 569 participants were evaluated in the meta-analysis. No significant heterogeneity was found between studies (*I*^2^=26%; *P*=.23; Figure 3 [33-41,43-45]). The intervention group had a significant QOL improvement compared to the control group (SMD 0.37, 95% CI 0.18-0.57; *P*<.001). In addition, the exclusion of any single study at one time did not change the pooled results markedly.

#### Effects on Sleep

Five studies investigated the impact of app- and website-based MBIs on sleep quality using 3 assessment tools: the 8-item PROMIS Sleep Disturbance scale [33], the Insomnia Severity Index [37,44], and the Pittsburgh Sleep Quality Index [35,36]. A higher score indicated a worse sleep quality. Moderate heterogeneity of effect sizes was observed (*I*²=58%; *P*=.05; Figure 3). Grouping the studies by type of technology, scale, and intervention type did not resolve heterogeneity, so a random effects model was chosen to pool the results. The result revealed that app- and website-based MBIs could alleviate patients’ sleep issues, with a statistical difference (SMD −0.36, 95% CI −0.72 to −0.01; *P*=.04). Only 1 outlier was detected [36]. After omitting the studies from the analysis, the effect size dropped to an SMD of −0.25 (95% CI −0.54 to 0.04; *P*=.09), and heterogeneity reduced substantially (*I*^2^=38%). The possible reason for this change may be attributed to the fact that small sample sizes tend to yield more pronounced effects.

#### Effects on FCR

A total of 3 studies measured FCR; the pooled data included 224 participants. Two FCR measures were used: FCRI [32,41] and the Short-Form FCRI [38]. A higher score indicated a higher level of FCR. There is great heterogeneity among the studies (*I*^2^=86%; *P*=.009; Figure 3). After the data of the study by Russell et al [41] are eliminated by the method of eliminating one by one, there is significantly lower heterogeneity (*I*^2^=0%; *P*=.70). This may be due to Russell et al [41] presurveying patients with cancer so that the intervention on FCR was more effective. The results showed that the difference between the network-based MBIs and the control group was not statistically significant (SMD −0.30, 95% CI −1.04 to 0.44; *P*=.39).

#### Effects on PTG

Two studies examined the effect of app- and website-based MBIs on PTG, with a total of 134 participants. The measurement tool exclusively used across 2 studies to assess PTG was the Posttraumatic Growth Inventory [33,45]. Higher scores indicated greater PTG. No significant heterogeneity was found between studies (*I*^2^=0%; *P*=.38; Figure 3). We found that app- and website-based MBIs did not lead to a significant increase in PTG score (SMD 0.08, 95% CI −0.26 to 0.42; *P*=.66).

#### Effects on Anxiety

**Figure 4:**
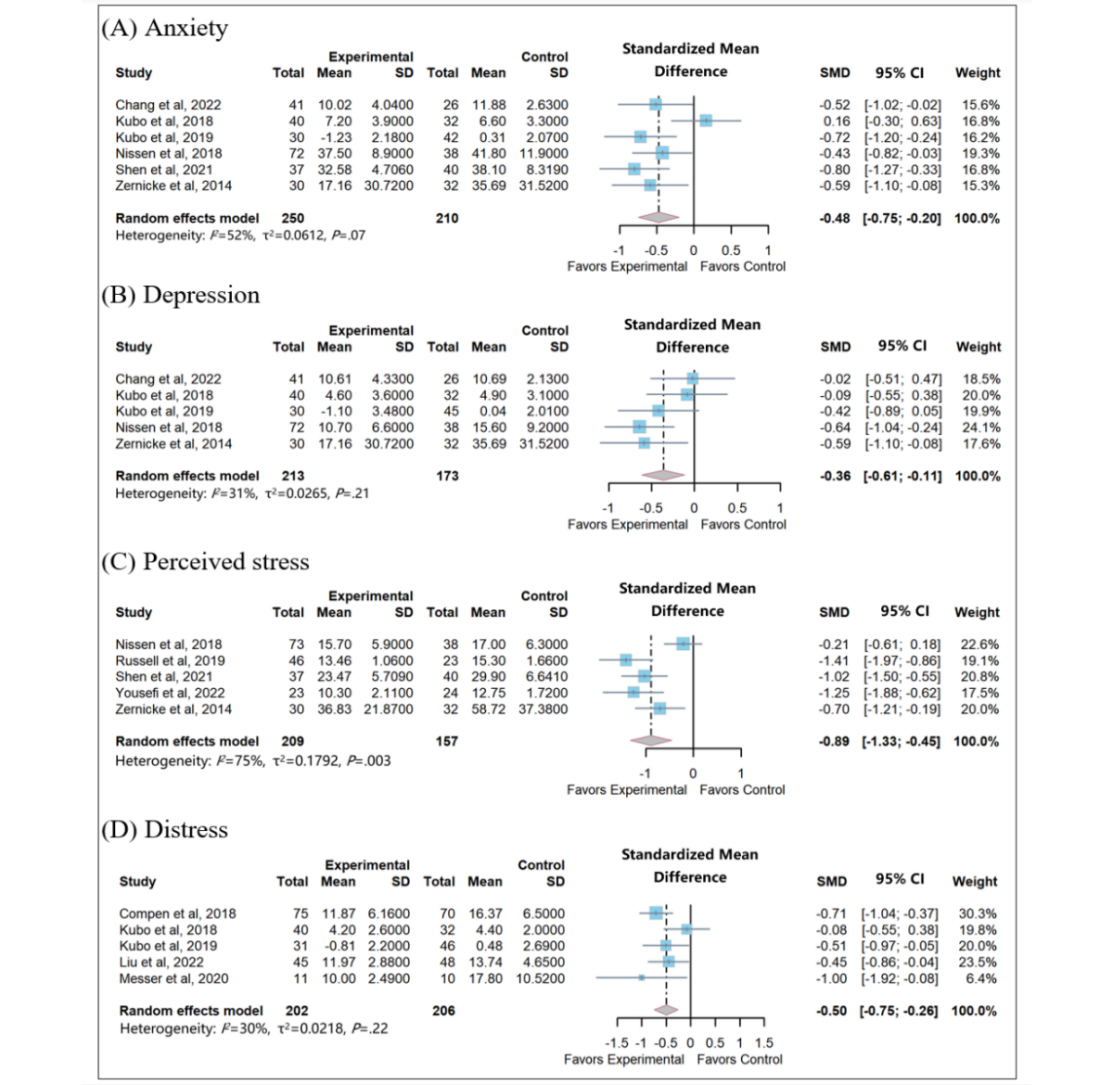
Meta-analysis on (A) anxiety, (B) depression, (C) perceived stress, and (D) distress. SMD: standardized mean difference.

Anxiety levels were assessed in 6 studies using 5 validated scales. These scales include the Hospital Anxiety and Depression Scale (HADS) [33,34], the Depression Anxiety Stress Scale Depression Inventory [31], the State-Trait Anxiety Inventory Y-Form [37], the Self-Rating Anxiety Scale [42], and the Profile of Mood States [45]. Higher scores on these scales indicated elevated levels of anxiety. Meta-analysis showed that app- and website-based MBIs lead to a significant reduction in anxiety (SMD −0.48, 95% CI −0.75 to −0.20; *P*<.001; Figure 4 [31-37,41,42,44,45]). Moderate heterogeneity was found between studies (*I*^2^=52%; *P*=.07). Grouping the studies by type of technology and intervention duration did not resolve heterogeneity (Table 2). Furthermore, when we examined subgroups based on sex, we found that studies including female participants had a significantly larger pooled effect size (SMD −0.67, 95% CI −1.01 to −0.33; *P*<.001) than the studies including both male and female participants (referred to as the mixed-gender subgroup; SMD −0.39, 95% CI −0.76 to −0.02; *P*=.04; Figure 4). The differences across these 2 subgroups were statistically nonsignificant (χ^2^_1_=1.2; *P*=.28).

**Table 2:** Subgroup analyses of anxiety, perceived stress, and sleep.

Subgroup and stratification				Studies, n (%)		SMD^a^ (95% CI)		*P* value for heterogeneity		*I* ^2^		*P* value for pooled results		*P* value for interaction	
**Anxiety (n=6)**															
	**Technology**														.54
		Website	3 (50)		−0.57 (−0.82 to −0.31)		.47		0		.0001				
		App	3 (50)		−0.38 (−0.93 to 0.18)		.02		74		.18				
	**Intervention duration**														.43
		<8 wk	2 (33)		−0.62 (−0.97 to−0.27)		.57		0		<.001				
		≥8 wk	4 (67)		−0.41 (−0.81 to −0.01)		.03		67		.04				
	**Sex**														.28
		Female	2 (33)		−0.67 (−1.01 to −0.33)		.41		0		<.001				
		Mixed	4 (67)		−0.39 (−0.76 to −0.02)		.05		62		.04				
	**Intervention type**														.67
		MBCR^b^	2 (33)		−0.70 (−0.05 to −0.36)		.55		0		<.001				
		MBCT^c^	1 (17)		−0.43 (−0.82 to 0.03)		—^d^		—		.04				
		MBIs^e^	2 (33)		−0.28 (−1.14 to 0.59)		.01		85		.53				
		MBSR^f^	1 (17)		−0.52(−1.02 to −0.02)		—		—		.04				
	**Study quality**														.07
		Unclear risk^g^	5 (83)		−0.43 (−0.74 to −0.12)		.06		56		.007				
		High risk^h^	1 (17)		−0.72 (−1.20 to −0.24)		—		—		.004				
**Perceived stress (n=5)**															
	**Technology**														.68
		Website	4 (80)		−0.87 (−1.44 to −0.29)		.002		80		.003				
		App	1 (20)		−1.02 (−1.50 to −0.55)		—		—		<.001				
	**Intervention type**														<.001
		MBCR	3 (60)		−0.96 (−1.27 to−0.66)		.39		0		<.001				
		MBCT	1 (20)		−0.21 (-0.61 to 0.18)		—		—		.29				
		MBIs	1 (20)		−1.41 (−1.97 to −0.86)		—		—		<.001				
**Sleep (n=5)**															
	**Scale**														.33
		PROMs^i^	1 (20)		−0.09 (−0.55 to 0.38)		—		—		.72				
		PSQI^j^	2 (40)		−0.78 (−1.58 to 0.02)		.11		60		.05				
		ISI^k^	2 (40)		−0.23 (−0.83 to 0.36)		.09		65		.44				
	**Technology**														.20
		Website	3 (60)		−0.52 (−1.22 to 0.19)		.02		75		.15				
		App	2 (40)		−0.02 (−0.32 to 0.28)		.70		0		.91				
	**Intervention duration**														.16
		<8 wk	2 (40)		−0.78 (−1.58 to −0.02)		.11		60		.05				
		≥8 wk	3 (60)		−0.16 (−0.49 to 0.17)		.23		32		.35				
	**Study quality**														.11
		Unclear risk	4 (80)		−2.03 (−2.93 to −1.13)		.12		49		<.001				
		High risk	1 (20)		0.20 (−2.93 to 2.79)		—		—		.88				

^a^SMD: standardized mean difference.

^b^MBCR: mindfulness-based cancer recovery.

^c^MBCT: mindfulness-based cognitive therapy.

^d^Not applicable.

^e^MBI: mindfulness-based intervention.

^f^MBSR: mindfulness-based stress reduction.

^g^Unclear risk: unclear risk of bias for one or more key domains.

^h^High risk: high risk of bias for one or more key domains.

^i^PROM: patient‐reported outcome measure.

^j^PSQI: Pittsburgh Sleep Quality Index.

^k^ISI: Insomnia Severity Index.

#### Effects on Depression

Depression was assessed across 5 studies using various standardized instruments. These included the Depression Anxiety Stress Scale-21 [31], HADS [33,34], Beck Depression Inventory [37], and Profile of Mood States [45]. Elevated levels of depression were indicated by higher scores on these scales. The pooled data included 384 participants and showed a significant difference in improvement between the intervention and control groups (SMD −0.36, 95% CI −0.61 to −0.11; *P*=.005; Figure 4). Moderate heterogeneity of effect sizes was observed (*I*^2^=31%; *P*=.21). In the sensitivity analysis using the one-study-out method, we found that the pooled estimates were not significantly altered when any 1 study was omitted in turn. The range of *P* values obtained varied from .0001 to .03, indicating that the summary effect size is robust.

#### Effects on Perceived Stress

A total of studies investigated the effects of app- and website-based MBIs on stress. Four distress measures were used: the Perceived Stress Scale [35,37,41], the Chinese version of the Perceived Stress Scale [42], the Depression and Stress Scale [44], and the Calgary Symptoms of Stress Inventory [45]. A total of 5 studies including 366 participants were evaluated in the meta-analysis. The data from 1 study was not pooled because the mean values and SD of outcomes were not reported [35], and between-study heterogeneity was found (*I*^2^=75%; *P*=.003; Figure 4). This meta-analysis revealed a reduction in stress of −0.89 (95% CI −1.33 to −0.45) when comparing the intervention group to the control group at the postintervention stage.

To further explore the potential sources of heterogeneity, we conducted subgroup analyses by type of technology and intervention type (Table 2). The 2 studies using apps (SMD −1.02, 95% CI −1.50 to −0.55; *I^2^*=0) were found to have low heterogeneity, whereas the 3 studies based on website-based technologies (SMD −0.87, 95% CI −1.44 to −0.29; *P*=.002) exhibited higher heterogeneity. After conducting sensitivity analysis and eliminating 1 study at a time, the exclusion of the study by Nissen et al [37] resulted in significantly lower heterogeneity (I*^2^*=21%; *P*=.28). One possible reason is that the study by Nissen et al [37], which offered internet-delivered MBCT as a routine based on a screening procedure, may have included less motivated participants compared to studies with self-referral. In addition, Nissen et al [37] used a lower cutoff value for screening the study population, which could have resulted in a floor effect.

#### Effects on Distress

In the analysis of distress (involving 5 studies), HADS [32,35,36] and the National Comprehensive Cancer Network Distress Thermometer [33,34] were used to assess the current distress level. Low heterogeneity was found between studies (*I*^2^=30%; *P*=.22; Figure 4), and the random effects model indicated that app- and website-based MBIs were associated with reduced distress levels in patients with cancer (SMD −0.50, 95% CI −0.75 to −0.26; *P*<.001).

#### Subgroup Analysis

Table 2 displays the results of subgroup analyses that were conducted to investigate the heterogeneity in the association between anxiety, perceived stress, and sleep in the context of MBIs. To explain the variability in the effects of mindfulness, we examined various moderating variables, such as technology, sex, intervention type, intervention duration, study quality, and scale. No statistically significant variables were found in the subgroup analysis of anxiety and sleep, whereas the type of intervention (*P*<.001) was a significant moderating variable for perceived stress

#### Publication Bias

Funnel plots and statistical tests were not performed as any of the outcomes had at least 10 studies to ensure sufficient power in detecting asymmetry [46]. However, we reduced the possibility of publication bias by conducting a thorough search across multiple databases to identify published studies [47].

#### RE-AIM Framework

Details of the RE-AIM Framework assessment are presented in Multimedia Appendix 3 [31-45]. Of the 15 studies, 14 (93%) reported 13% to 92% of eligible patients. Efficacy (effect size and 95% CI of primary outcome) was reported in 33% (5/15) of the studies [33,35,37,39,40] (Cohen *d* or *η*^2^). For adoption barriers, health professionals or researchers conducted recruitment for all studies, and 53% (8/15) of the studies [35-38,41-44] recruited participants in person (hospital and cancer center). For implementation, intervention adherence ranged from 59% to 100% of participants completing all scheduled components. Dropouts of most complex interventions ranged from 0% to 48%, with 40% (6/15) of the studies [31,38-40,42,45] having <10% dropouts. The cost was reported in 4 studies [33,34,39,40], including the paid app (priced at US $77 for 6 months and US $69.99 for 12 months) and the app already publicly available. In total, 46% (7/15) of the studies [35,37-40,43,44] reported maintenance of results, and 46% (7/15) of the studies [35,37-40,43,44] sustained results for 1 to 9 months. Four studies [33,34,39,40] explicitly reported on the potential for the interventions to remain accessible or whether there were plans for their continued implementation.

## Discussion

### Principal Findings

The objective of this study is to assess the effectiveness of MBIs in improving the mental health and QOL of patients with cancer. We discovered that patients’ QOL can be greatly enhanced by app- and website-based MBIs, which also significantly lowers psychological distress, sleep problems, anxiety, depression, and perceived stress. This systematic review of meta-analyses and the RE-AIM framework demonstrate that app- and website-based interventions have a wide range of effects and are highly used by different (international and multilingual) patients with cancer. However, the use and accessibility of app- and website-based MBIs for patients with cancer have been constrained because of service fees and patient mobility limitations [48]; app- and website-based MBIs are mainly conducted in high-income countries. The possible explanation is the distinction between communication and economy; some high-income countries may have national health services in place to promote app- and website-based MBIs, whereas developing nations may not. Study shows that in many low- and middle-income countries, the accessibility of evidence-based mental health treatments remains limited [49]. The time commitment, teacher shortage, and high cost of classic mindfulness interventions may have hindered efforts to spread the associated benefits to individuals in developing countries [50]. For instance, Indonesia has yet to implement evidence-based internet-based mindfulness therapy, emphasizing the need for expanding evidence-based mental health interventions in resource-constrained settings.

The results of this study suggest that app- and website-based MBIs are effective in improving QOL and reducing anxiety and depressive symptoms in patients with cancer, which is consistent with previous meta-analyses [18,20]. A possible explanation for this is that app- and website-based MBIs can alleviate negative emotions, enhance positive emotions, and increase mindfulness skills among patients with cancer, as elaborated by previous research [51]. Moreover, the sleep quality of patients with cancer also improved after MBIs. This outcome may be attributed to the inclusion of techniques in the program that target sleep difficulties [7] and the nonjudging aspect of mindfulness, which can enhance sleep quality by mitigating stress and everyday tensions. Previous studies [52] have confirmed the moderate effect of mindfulness interventions on sleep quality, which suggests that the use of app- and website-based MBIs to manage QOL and sleep in patients with cancer should be further supported.

App- and website-based MBIs have shown potential in helping patients with cancer develop emotional regulation skills and cope with the distress associated with diagnosis and treatment [53]. It makes patients feel better emotionally and physically and helps patients with cancer reduce their psychological distress [54]. Incorporating MBIs into oncological treatment can promote emotional and physical well-being and alleviate psychological distress [55]. MBIs have been found to regulate biological variables associated with stress [56], such as immune function, hypothalamic-pituitary-adrenal regulation, and autonomic nervous system activity, thereby reducing pressure on patients. The data from this review showed that MBCR appeared to be particularly effective in reducing perceived stress, whereas MBCT was not effective in reducing stress after the intervention [51]. This finding was unexpected, given that many previous studies have suggested the effectiveness of MBCT in reducing stress [57]. However, because of the limited number of included studies, it is difficult to draw definitive conclusions regarding the comparative effectiveness of different MBIs.

However, although not statistically significant, app- and website-based MBIs can improve the level of PTG and FCR in patients with cancer. FCR is one of the most common problems of survivors of cancer, and it has been known that FCR can persist throughout the treatment and survival trajectory [58]; thus, specific intervention is needed for survivors of cancer who have clinically significant FCR. Previous meta-analysis showed that cognitive therapy and mindfulness exercises are very suitable for combating FCR [59]. Numerous psychological and behavioral mechanisms of change within mindfulness interventions have been suggested, encompassing acceptance, emotion regulation skills, and the reduction of ruminative thoughts [60]. The meta-analysis by Gu et al [61] provided empirical confirmation that rumination significantly mediates the impact of MBIs on mental health outcomes. In addition, the study by Butow et al [62] identified rumination as a crucial psychological mechanism associated with FCR. Therefore, the study suggests that the effectiveness of mindfulness interventions in addressing the FCR may be attributed to their potential to improve patients’ levels of rumination. The improved PTG observed in this study may be explained by the systematic training in moment-by-moment awareness, and MBIs focus on viewing thoughts and feelings as mental events [63]. Such a *decentered* relationship enables a perception of mental events as aspects of experience moving through awareness, showing that mindfulness practice supports personal growth and transformation.

In this study, it was observed that short-term MBIs with a duration of <8 weeks exhibited a larger effect size concerning the outcomes of anxiety and sleep. In the study by Wang et al [43], short-term MBIs were found to be more effective in improving physical health compared to long-term MBIs, and interventions lasting <8 weeks demonstrated a greater effect size, possibly attributed to the increased participant engagement resulting from the shorter intervention duration and simplified intervention complexity. Shorter interventions may be more feasible and acceptable for patients with cancer who are dealing with a range of physical and emotional challenges [64]. Future research should aim to replicate and expand on these results, including investigating the optimal duration and timing of app- and website-based MBIs for patients with cancer.

### Recommendations for Future Research

To the best of our knowledge, this study represents the first meta-analysis using the RE-AIM framework, systematically reviewing and synthesizing the effectiveness of MBIs for patients with cancer across various types of interventions. By accurately reporting the RE-AIM dimensions, this study seeks to enhance the replicability and universality of mindfulness interventions in oncology settings. Our assessment of app- and website-based MBIs for patients with cancer, conducted within the framework of RE-AIM, reveals that the participation rates of eligible patients range from 13% to 92%. The calculated median participation rate, at 67% (IQR 47.5%-82%), emphasizes the effectiveness of the interventions in reaching a substantial portion of the target population. However, only a minority of studies reported on efficacy, which limited our ability to draw conclusions on overall effectiveness. Recruitment was primarily conducted by health professionals or researchers, and more than half of the studies (8/15, 53%) recruited participants in person, potentially limiting generalizability. Intervention adherence was generally high, but dropout rates varied widely, indicating that certain interventions may be more challenging for some patients. Cost was reported in only a few studies (4/15, 27%), with implications for accessibility. Long-term effects were reported in more than half of the studies (7/15, 47%), highlighting the need for further research. This study underscores the importance of considering the RE-AIM framework in the implementation and evaluation of these interventions. Further research is needed to fully understand their potential benefits and limitations in real-world settings.

Internet-based interventions have previously been shown to be effective for anxiety disorders and fear-related disorders and have achieved the same effect as face-to-face treatment [65]. Consistent with the results of this study, delivery via the internet, group, or app is feasible and effective. Our results suggest that among forms of online MBIs for patients with cancer, the most widely studied type was website-based interventions. This observation is in line with an analysis conducted in recent years [66], which indicated that the most widely studied type of telehealth for patients with breast cancer was website-based interventions. Website-based MBIs may offer more content, functionality, and instruction than app-based interventions, which may enhance user engagement, learning, and practice of positive thinking skills [67,68]. Website-based MBIs had higher completion rates and lower attrition rates compared to app-based interventions, which may be due to factors such as convenience, accessibility, engagement, and personalization [69]. Finally, in our review, a website-based study [41] that greatly improved FCR and stress highlighted the sustainability and self-management of the intervention and enabled flexible navigation by accessing website content according to user preferences. Therefore, website-based MBIs may offer more opportunities for personalization and tailoring interventions to individual needs.

In our analysis, 53% (8/15) of the studies implemented a weekly or daily reminder system through various channels, such as email, text messages, apps, or smartphone notifications, to facilitate app- and website-based MBIs. However, the prevalence of reminder systems in the studies under review is relatively limited (7/15, 47%), a finding consistent with the investigation by Matis et al [19]. Matis et al conducted a systematic evaluation in this field, discussing the limited prevalence of reminder systems in reviewed studies and highlighting the current lack of direct comparisons between interventions with and without reminders. In addition, the study found that the frequency of reminders was positively associated with the magnitude of the intervention effect [70]. Consequently, to promote patient involvement in app- and website-based MBIs, it is vital to set reminders [67]. Some studies have also set up expert feedback, answers, and a variety of supervision methods to avoid reduced patient compliance. Therefore, app- and website-based MBIs can enhance engagement using features such as reminders, feedback, personalization, and facilitator-led components. However, it is important to note that the specific frequency, timing, and content of the reminders may vary depending on the individual and the context of the intervention. Our study results reveal heterogeneity in the types, frequencies, and content of reminder systems, preventing the establishment of specific standards for their effectiveness. Despite the evident practicality of reminder systems, a more comprehensive investigation into their types, frequencies, and effectiveness is imperative within the context of app- and website-based MBIs.

This systematic review found that most app- and website-based interventions have adopted online classrooms; application-based measures to implement mindfulness interventions; and multicomponent interventions that include audio, video, and documents. However, the study did not clarify which factors affect behavioral changes. Despite these differences, 67% (10/15) of the interventions are designed specifically for the cancer population and provide customizable interventions. For example, as demonstrated by Wang et al [43], a pilot website-based MBIs was conducted for patients with cancer; this is an adapted version of MBSR specifically tailored for individuals dealing with cancer-related stressors. The MBCR program retains the core principles and practices of MBSR while integrating specific intervention materials to address challenges associated with cancer, such as common experiences related to cancer, sleep issues, pain, and FCR, which is greatly beneficial for improving the physical and mental symptoms of patients with cancer. MBCR will provide a platform for patients with cancer to engage in discussions and address challenges related to cancer. Future app- and website-based MBIs should take into account the characteristics of patients and determine which intervention plan is most suitable for patients with cancer, emphasizing feedback sessions and communication with therapists to enable patients to learn self-management and make intervention plans sustainable.

### Limitations

Although this review summarizes international RCTs for various outcomes, there are limitations. First, because the research results are measured by various tools, it may hinder the comparability of research outcomes. Second, in the 15 trials, there are differences in the personnel, duration, and methods of app- and website-based MBIs in various studies. Patients included in these studies have different characteristics. Third, The inability to access or adequately translate studies in languages other than English and Chinese may introduce bias into the selection process, potentially limiting the comprehensiveness of the findings. Finally, in the subgroup analysis, the study of each subgroup is limited, which may reduce the ability to draw conclusions on the differences in the consistency of intervention effects between subgroups. The abovementioned factors may lead to heterogeneity between studies, which is closely related to the summary results, so these results need to be interpreted carefully. Nevertheless, the meta-analysis included RCTs only and used a random effect model to pool results to give the most conservative estimates. In addition, subgroup analysis and sensitivity analysis were conducted and showed that the pooled estimations were relatively robust.

### Conclusions

This meta-analysis provides definite evidence regarding the efficacy of app- and website-based MBIs for patients with cancer. Our findings suggest that app- and website-based MBIs can be effective in improving OL, sleep, and mental health and can be integrated into stepped care in clinical practice. Future experiments should pay more attention to the development of intervention programs based on the wishes and characteristics of patients with cancer and study how to optimize interventions further and customize interventions based on individual physical and mental symptoms.

## Appendix

Multimedia Appendix 1 Search strategy.

Multimedia Appendix 2 Summary of the risk of bias of studies included in the systematic review.

Multimedia Appendix 3 RE-AIM (Reach, Effectiveness, Adoption, Implementation, and Maintenance) framework.

Multimedia Appendix 4 Preferred Reporting Items for Systematic Reviews and Meta-Analyses (PRISMA) checklist.

## Abbreviations

FCR: fear of cancer recurrence
FCRI: Fear of Cancer Recurrence Inventory
HADS: Hospital Anxiety and Depression Scale
MBCT: mindfulness-based cognitive therapy
MBI: mindfulness-based intervention
MBSR: mindfulness-based stress reduction
PRISMA: Preferred Reporting Items for Systematic Reviews and Meta-Analyses
PTG: posttraumatic growth
QOL: quality of life
RCT: randomized controlled trial
RE-AIM: Reach, Effectiveness, Adoption, Implementation, and Maintenance
SMD: standardized mean difference

## Data Availability

All data generated or analyzed during this study are included in this published article and its supplementary information files.
