# Supplementary material for: Effectiveness of Web-Based Mindfulness-Based Interventions for Patients With Cancer: Systematic Review and Meta-Analyses
Source: J Med Internet Res. 2024 Jun 25;26:e47704. doi: 10.2196/47704 (PMC11234071; doi:10.2196/47704)
Supplement: Multimedia Appendix 1 [file jmir_v26i1e47704_app1.doc]

**Multimedia Appendix 1. All examples of search strategies**

| **PubMed (n=310)** | | |
| --- | --- | --- |
| **#1** | (((Cancer[Title/Abstract] OR Oncology[Title/Abstract] OR Tumo*[Title/Abstract] OR  Carcinoma[Title/Abstract] OR Carcinomas[Title/Abstract] OR malignant[Title/Abstract] OR  malignanc*[Title/Abstract] OR neoplasms[Title/Abstract]) OR (carcinoma[MeSH Terms]))  OR (neoplasm[MeSH Terms])) | 4890309 |
| **#2** | (("Mindfulness"[Mesh] OR "Acceptance and Commitment Therapy"[Mesh] OR  "Meditation"[Mesh]) OR (mindful*[tiab] OR acceptance[tiab] OR meditation[tiab])) | 104422 |
| **#3** | intervention*[tiab] OR therap*[tiab] OR treatment*[tiab] OR program*[tiab] | 8550843 |
| **#4** | (((((((mhealth[Title/Abstract] OR eHealth[Title/Abstract] OR online[Title/Abstract] OR Internet*[Title/Abstract] OR web-based[Title/Abstract] OR Web page[Title/Abstract] OR computer*[Title/Abstract] OR Internet-delivered[Title/Abstract] OR software[Title/Abstract] OR apps[Title/Abstract] OR smartphone[Title/Abstract])) OR telemedicine[Title/Abstract] OR telehealth[Title/Abstract])) OR ("mhealth app"[Title/Abstract]) OR ("mhealth application"[Title/Abstract]) OR app[Title/Abstract] OR ("mobile app"[Title/Abstract]) OR ("mobile application"[Title/Abstract]) OR ("smartphone app"[Title/Abstract]) OR ("smartphone application"[Title/Abstract] OR (Telemedicine[MeSH Terms])OR (smartphone[MeSH Terms]) OR (Computers[MeSH Terms]) OR ("Mobile Applications"[MeSH Terms])OR (Internet-Based Intervention[MeSH Terms]))) | 940871 |
| **#5** | "randomized controlled trial"[pt] OR "controlled clinical trial"[pt] OR randomized[tiab] OR placebo[tiab] OR "drug therapy"[sh] OR randomly[tiab] OR trial[tiab] OR groups[tiab] | 5593813 |
| **#6** | #1AND#2AND#3AND#4AND#5 |  |

| **Web of Science (n=1675 )** | | |
| --- | --- | --- |
| **#1** | TS=(Cancer OR Oncology OR Tumo* OR Carcinoma OR Carcinomas OR malignant OR malignanc* OR neoplasms) | [8141423](https://www.webofscience.com/wos/alldb/summary/cc61415e-e1c1-4a1b-a377-01063311285a-6342375d/relevance/1) |
| **#2** | TS=(mindful* OR acceptance OR meditation) | [390456](https://www.webofscience.com/wos/alldb/summary/34c864b0-197e-4adb-91b9-4a1b90f4f14f-63426ba5/relevance/1) |
| **#3** | TS=(intervention* OR therap* OR treatment* OR program*) | [26473918](https://www.webofscience.com/wos/alldb/summary/20b0d416-22b3-48af-bffc-c21bf27e3d3c-634260e5/relevance/1) |
| **#4** | TS=(mhealth OR eHealth OR online OR Internet* OR web-based OR Web page OR computer* OR Internet-delivered OR software OR apps OR smartphone OR telemedicine OR telehealth OR "mhealth app" OR "mhealth application" OR app OR "mobile app" OR "mobile application" OR "smartphone app" OR "smartphone application"mhealth OR eHealth OR online OR Internet* OR web-based OR Web page OR computer* OR Internet-delivered OR software OR apps OR smartphone OR telemedicine OR telehealth OR "mhealth app" OR "mhealth application" OR app OR "mobile app" OR "mobile application" OR "smartphone app" OR "smartphone application" | [11205704](https://www.webofscience.com/wos/alldb/summary/84a889b8-77ab-4fd3-a4d1-96c602a22bb9-6342582d/relevance/1) |
| **#5** | TS=(random* OR trial OR RCT OR control*) | [27351444](https://www.webofscience.com/wos/alldb/summary/f44b3c9b-dc1c-4791-80ea-b30e78bcca08-634272da/relevance/1) |
| **#6** | #1AND#2AND#3AND#4AND#5 |  |

| **CINAHL Complete (n=76 )** | | |
| --- | --- | --- |
| **#1** | TI ( Cancer OR Oncology OR Tumo* OR Carcinoma OR Carcinomas OR malignant OR malignanc* OR neoplasms ) OR AB ( Cancer OR Oncology OR Tumo* OR Carcinoma OR Carcinomas OR malignant OR malignanc* OR neoplasms ) | 484399 |
| **#2** | AB ( mindful* OR acceptance OR meditation OR "Acceptance and Commitment Therapy" ) OR TI ( mindful* OR acceptance OR meditation OR "Acceptance and Commitment Therapy" ) | 26362 |
| **#3** | TI ( intervention* OR therap* OR treatment* OR program* ) OR AB ( intervention* OR therap* OR treatment* OR program* ) | 1334946 |
| **#4** | AB ( mhealth OR eHealth OR online OR Internet* OR "web-based" OR "Web page" OR computer* OR "Internet-delivered" OR software OR apps OR smartphone OR telemedicine OR telehealth OR "mhealth app" OR "mhealth application" OR app OR mobile app" OR "mobile application" OR "smartphone app" OR "smartphone application" ) OR TI ( mhealth OR eHealth OR online OR Internet* OR "web-based" OR "Web page" OR computer* OR "Internet-delivered" OR software OR apps OR smartphone OR telemedicine OR telehealth OR "mhealth app" OR "mhealth application" OR app OR mobile app" OR "mobile application" OR "smartphone app" OR "smartphone application" ) | 154319 |
| **#5** | TI ( random* OR trial OR RCT OR control* ) OR AB ( random* ORtrial OR RCT OR control* ) | 772136 |
| **#6** | #1AND#2AND#3AND#4AND#5 |  |

| **EMBASE (n=447)** | | |
| --- | --- | --- |
| **#1** | cancer:ti,ab,kw OR oncology:ti,ab,kw OR tumor:ti,ab,kw OR carcinoma:ti,ab,kw OR carcinomas:ti,ab,kw OR malignant:ti,ab,kw OR malignancy:ti,ab,kw OR neoplasms:ti,ab,kw | 4846057 |
| **#2** | mindful*:ti,ab,kw OR acceptance:ti,ab,kw OR meditation:ti,ab,kw OR 'mindfulness'/exp/mj OR ('acceptance'/exp/mj AND 'commitment therapy') OR 'meditation'/exp/mj | 136999 |
| **#3** | intervention*:ti,ab,kw OR therap*:ti,ab,kw OR treatment*:ti,ab,kw OR program*:ti,ab,kw | 11730677 |
| **#4** | mhealth:ti,ab,kw OR ehealth:ti,ab,kw OR online:ti,ab,kw OR internet*:ti,ab,kw OR 'web-based':ti,ab,kw OR 'web page':ti,ab,kw OR computer*:ti,ab,kw OR 'internet-delivered':ti,ab,kw OR software:ti,ab,kw OR apps:ti,ab,kw OR smartphone:ti,ab,kw OR telemedicine:ti,ab,kw OR telehealth:ti,ab,kw OR 'mhealth app':ti,ab,kw OR 'mhealth application':ti,ab,kw OR app:ti,ab,kw OR 'mobile app':ti,ab,kw OR 'mobile application':ti,ab,kw OR 'smartphone app':ti,ab,kw OR 'smartphone application':ti,ab,kw | 5763693 |
| **#5** | random*:ti,ab,kw OR trial:ti,ab,kw OR rct:ti,ab,kw OR control:ti,ab,kw | 1230759 |
| **#6** | #1AND#2AND#3AND#4AND#5 |  |

| **Cochrane library (n=1570)** | | |
| --- | --- | --- |
| **#1** | (Cancer OR Oncology OR Tumo* OR Carcinoma OR Carcinomas OR malignant OR malignanc* OR neoplasms):ti,ab,kw OR MeSH descriptor: [Carcinoma] explode all trees OR MeSH descriptor: [Neoplasms] explode all trees | 265359 |
| **#2** | MeSH descriptor: [Mindfulness] explode all trees OR MeSH descriptor: [Acceptance and Commitment Therapy] explode all trees OR MeSH descriptor: [Meditation] explode all trees OR (mindful* OR acceptance OR meditation):ti,ab,kw | 78474 |
| **#3** | (intervention* OR therap* OR treatment* OR program*):ti,ab,kw | 1409767 |
| **#4** | (mhealth OR eHealth OR online OR Internet* OR web-based OR Web page OR computer* OR Internet-delivered OR software OR apps OR smartphone OR telemedicine OR telehealth OR "mhealth app" OR "mhealth application" OR app OR "mobile app" OR "mobile application" OR "smartphone app" OR "smartphone application"mhealth OR eHealth OR online OR Internet* OR web-based OR Web page OR computer* OR Internet-delivered OR software OR apps OR smartphone OR telemedicine OR telehealth OR "mhealth app" OR "mhealth application" OR app OR "mobile app" OR "mobile application" OR "smartphone app" OR "smartphone application"):ti,ab,kw OR MeSH descriptor: [Telemedicine] explode all trees OR MeSH descriptor: [Smartphone] explode all trees OR MeSH descriptor: [Computers] explode all trees OR MeSH descriptor: [Mobile Applications] explode all trees OR MeSH descriptor: [Internet-Based Intervention] explode all trees | 123558 |
| **#5** | (random* OR trial OR RCT OR control*):ti,ab,kw | 1518760 |
| **#6** | #1AND#2AND#3AND#4AND#5 |  |

| **SCOPUS(n=175)** | | |
| --- | --- | --- |
| **#1** | TITLE-ABS-KEY ( cancer OR oncology OR tumo* OR carcinoma OR carcinomas OR malignant OR malignanc* OR neoplasms ) | 5969557 |
| **#2** | TITLE-ABS-KEY ( mindful* OR acceptance OR meditation) | 351140 |
| **#3** | TITLE-ABS-KEY ( intervention* OR therap*OR treatment* OR program* ) | 16871222 |
| **#4** | TITLE-ABS-KEY ( mhealth OR ehealth OR online OR internet* OR web-based OR web AND page OR computer* OR internet-delivered OR software OR apps OR smartphone OR telemedicine OR telehealth OR "mhealth app" OR "mhealth application" OR app OR "mobile app" OR "mobile application" OR "smartphone app" OR "smartphone application" mhealth OR ehealth OR online OR internet* OR web-based OR web AND page OR computer* OR internet-delivered OR software OR apps OR smartphone OR telemedicine OR telehealth OR "mhealth app" OR "mhealth application" OR app OR "mobile app" OR "mobile application" OR "smartphone app" OR "smartphone application" ) | 560554 |
| **#5** | TITLE-ABS-KEY ( random* OR trial OR rct OR control*) | 190114 |
| **#6** | #1AND#2AND#3AND#4AND#5 |  |

| **PsycINFO****(n=81)** | | |
| --- | --- | --- |
| **#1** | TI ( Cancer OR Oncology OR Tumo* OR Carcinoma OR Carcinomas OR malignant OR malignanc* OR neoplasms ) OR AB ( Cancer OR Oncology OR Tumo* OR Carcinoma OR Carcinomas OR malignant OR malignanc* OR neoplasms ) OR KW ( Cancer OR Oncology OR Tumo* OR Carcinoma OR Carcinomas OR malignant OR malignanc* OR neoplasms ) OR DE (carcinoma OR neoplasm) | 92386 |
| **#2** | TI ( mindful* OR acceptance OR meditation ) OR AB ( mindful* OR acceptance OR meditation ) OR KW ( mindful* OR acceptance OR meditation ) ) OR DE ( "Mindfulness” OR "Acceptance and Commitment Therapy OR Meditation" ) | 81937 |
| **#3** | TI ( intervention* OR therap* OR treatment* OR program* ) OR AB ( intervention* OR therap* OR treatment* OR program* ) OR KW ( intervention* OR therap* OR treatment* OR program* ) | 1513188 |
| **#4** | TI ( mhealth OR eHealth OR online OR Internet* OR "web-based" OR "Web page" OR computer* OR "Internet-delivered" OR software OR apps OR smartphone OR telemedicine OR telehealth OR "mhealth app" OR "mhealth application" OR app OR mobile app" OR "mobile application" OR "smartphone app" OR "smartphone application" ) OR AB ( mhealth OR eHealth OR online OR Internet* OR "web-based" OR "Web page" OR computer* OR "Internet-delivered" OR software OR apps OR smartphone OR telemedicine OR telehealth OR "mhealth app" OR "mhealth application" OR app OR mobile app" OR "mobile application" OR "smartphone app" OR "smartphone application" ) OR DE ( Telemedicine OR Computers OR smartphone OR "Mobile Applications" OR "Internet-Based Intervention" )) | 281487 |
| **#5** | AB ( random* OR trial OR RCT OR control* ) OR TI ( random* OR trial OR RCT OR control* ) OR KW ( random* OR trial OR RCT OR control* ) OR DE ("Clinical trials" OR "Treatment Effectiveness Evaluation") | 1002145 |
| **#6** | #1AND#2AND#3AND#4AND#5 |  |

| **SinoMed (n=15)** | | |
| --- | --- | --- |
| **#1** | ("癌症"[常用字段] OR "肿瘤"[常用字段] OR "瘤"[常用字段] OR "恶性肿瘤"[常用字段] OR "良性肿瘤"[常用字段] OR "肿瘤"[主题词]) OR ("肿瘤"[常用字段] OR "瘤"[常用字段] OR "癌症"[常用字段] OR "恶性肿瘤"[常用字段] OR "良性肿瘤"[常用字段] OR "肿瘤"[主题词])) | 91532 |
| **#2** | ("正念"[常用字段:智能] OR "接纳承诺疗法"[常用字段:智能] OR "冥想"[常用字段:智能]) | 12043 |
| **#3** | "网络"[常用字段:智能] OR "电子"[常用字段:智能] OR "移动"[常用字段:智能] OR "远程"[常用字段:智能] OR "智能"[常用字段:智能] OR "手机"[常用字段:智能]) | 38512 |
| **#4** | #1AND#2AND#3 |  |
