# Supplementary material for: Effectiveness of Web-Based Mindfulness-Based Interventions for Patients With Cancer: Systematic Review and Meta-Analyses
Source: J Med Internet Res. 2024 Jun 25;26:e47704. doi: 10.2196/47704 (PMC11234071; doi:10.2196/47704)
Supplement: Multimedia Appendix 2 [file jmir_v26i1e47704_app2.doc]

Multimedia Appendix 2. Cochrane Risk of Bias Supporting Evidence

| Author Year | Random sequence generation  (selection bias) | | Allocation concealment (selection bias) | | Blinding of participants and personnel  (performance bias) | | Blinding of outcome  assessment  (detection bias) | | Incomplete outcome data  (attrition bias) | | Selective reporting  (reporting bias) | | Other bias | Within a study |
| --- | --- | --- | --- | --- | --- | --- | --- | --- | --- | --- | --- | --- | --- | --- |
|  | **Judgment** | **Supporting evidence** | **Judgment** | **Supporting evidence** | **Judgment** | **Supporting evidence** | **Judgment** | **Supporting evidence** | **Judgment** | **Supporting evidence** | **Judgment** | **Supporting evidence** | **Judgment** | **Judgment** |
| Shen et al [31], 2021 | low^a^ | Random number table | low | Opaque, sealed envelope | unclear^b^ | The author did not state the use of blinding method | unclear | The author did not state the use of blinding method | low | Attrition and reasons for attrition  reported and similar across  groups | unclear | The study protocol is not available | low | unclear^c^ |
| Zernicke et al [45], 2014 | low | Computer-based random number generation program | low | Remained blind to group allocation until after completion of baseline | unclear | The nature of the group assignment and intervention did not allow for masking of participants | low | Primary investigators remained blind to participant status | low | Attrition and reasons for attrition  reported and similar across  groups | low | Study protocol and trial  registration available and all  pre-specified outcomes  reported in pre-specified way | unclear | unclear |
| Yousefi et al [32], 2022 | low | Drawing lots | unclear | Insufficient information | unclear | The author did not state the use of blinding method | unclear | The author did not state the use of blinding method | unclear | The author did not describe the lost interview case | unclear | The study protocol is not available | low | unclear |
| Wang et al [44], 2022 | low | A list of computer-generated random numbers | low | Delivering an opaque, sealed envelope to each participant | unclear | unclearecause of the nature of the intervention, participants could not be blinded | low | The research assistants were blinded to each participant’s group allocation | low | Attrition rates (low) and reasons  were reported and similar  between groups;  The intention-to-treat analysis was applied | unclear | The study protocol is not available | low | unclear |
| Russell et al [43], 2019 | low | Generated by a Secure Sockets Layer | low | Study personnel did not have access to the sequences and were blinded to group assignment | unclear | Participants were unblinded to group assignment, as the intervention did not allow for blinding | low | All questionnaires will be completed online, which will reduce the influence of researcher bias | low | Attrition rates (low) and reasons  were reported and similar  between groups  All analyses were undertaken on an intention-to-treat basis | low | Study protocol and trial  registration available and all  pre-specified outcomes  reported in pre-specified way | low | unclear |
| Rosen [35], 2017 | low | REDCap randomization tool | high^d^ | Group assignment was not blinded. | unclear | Due to the nature of the intervention, group assignment was not blinded | unclear | The author did not state the use of blinding method | low | No participants were removed from the dataset due to missing values | unclear | The study protocol is not available | low | high^e^ |
| Rosen et al [36], 2018 | low | REDCap randomization module | high | Group assignment was not blinded | unclear | Due to the nature of the intervention, group assignment was not blinded | unclear | The author did not state the use of blinding method | high | Attrition was higher among intervention compared to control group; Intent to treat analyses | unclear | The study protocol is not available | low | high |
| Peng et al [33], 2022 | low | Patients were randomly divided into the group | unclear | Not mention | unclear | The author did not state the use of blinding method | unclear | The author did not state the use of blinding method | low | Attrition and reasons for attrition  reported and similar across  groups | unclear | The study protocol is not available | low | unclear |
| Nissen et al [34], 2018 | low | Computerized randomization | low | Computer-generated  randomization list concealed | unclear | The author did not state the use of blinding method | unclear | The author did not state the use of blinding method | low | Attrition rates (low) and reasons  were reported and similar  between groups.intention‐to‐treat analyses were conducted | high | Additional variables, not reported on in the present paper, included measures of mindfulness, self‐compassion, distress, and therapeutic alliance | unclear | high |
| Messer et al [42], 2020 | low | Website algorithm | low | Researchers were blind to participant condition | low | Provided blinded treatment to rule out the effects of non-specific group factors | low | Provided blinded treatment to rule out the effects of non-specific group factors | low | Attrition and reasons for attrition  reported and similar across  groups | unclear | The study protocol is not available | unclear | unclear |
| Liu et al [41], 2022 | low | Research Randomizer (version 4.0) | low | The outcome assessors were masked to the participant’s group allocation | unclear | The author did not state the use of blinding method | low | The person in charge of data analysis did not understand the grouping | low | Attrition rates (low) and reasons  were reported and similar  between groups; Intention-to-treat analysis method was used to analyse the data | unclear | secondary outcomes: Mindfulness level, pain,acceptance, Self adjusting not be reported | low | unclear |
| Kubo et al [37], 2019 | unclear | Stratification on clinic size | unclear | Insufficient information | unclear | The nature of the group assignment and intervention did not allow for masking of participants or clinicians | low | Main researchers know nothing about the status of the participants | low | Attrition rates (low) and reasons  were reported and similar  between group; Intent‐to‐treat basis | unclear | The study protocol is not available | low | unclear |
| Kubo et al [40], 2018 | low | Simple balanced blocked randomization, stratified by facility | low | Sequentially numbered opaque envelopes filled by research personnel | unclear | The author did not state the use of blinding method | unclear | The author did not state the use of blinding method | low | Attrition and reasons for attrition  reported and similar across  groups | unclear | The study protocol is not available | unclear | unclear |
| Compen et al [39], 2018 | low | Computer-generated random numbers sequence | low | Randomisation will occur using block randomisation undertaken by the study manage | unclear | The author did not state the use of blinding method | low | Project staff will be blinded to study condition,the standardized psychiatric interviews were conducted assistants who were blinded to treatment allocation | low | Missing continuous outcomes were imputed with automatic multiple imputation on the basis of linear regression | high | Additional variables, not reported on in the present paper, included FACT-FS, SF-36 | low | high |
| Chang et al [38], 2022 | unclear | Insufficient  information  to permit  judgement | unclear | Insufficient  information | unclear | Insufficient  information | unclear | Insufficient  information | low | Attrition and reasons for attrition  reported and similar across  groups | low | Study protocol and trial  registration available and all  pre-specified outcomes  reported in pre-specified way | unclear | unclear |

^a^Low risk of bias within domains: Plausible bias unlikely to seriously alter the results.

^b^Unclear risk of bias within domains: Plausible bias that raises some doubt about the results.

^c^Unclear risk of bias within a study: Unclear risk of bias for one or more key domains.

^d^High risk of bias within domains: Plausible bias that seriously weakens confidence in the results.

^e^High risk of bias within a study: High risk of bias for one or more key domains.
