# Supplementary material for: Effectiveness of Web-Based Mindfulness-Based Interventions for Patients With Cancer: Systematic Review and Meta-Analyses
Source: J Med Internet Res. 2024 Jun 25;26:e47704. doi: 10.2196/47704 (PMC11234071; doi:10.2196/47704)
Supplement: Multimedia Appendix 3 [file jmir_v26i1e47704_app3.doc]

Multimedia Appendix 3. RE-AIM^a^ Framework

| Author,  Year,  Country,  Citation | Reach n  randomised/ N Eligible  (%) | Efficacy  Primary outcome  Effect size (95%CI)^b^ | Adoption Recruitment personnel and  setting | Implementation Adherence to intervention | Implementation Dropouts  n/N (%): | Implementation Most complex  intervention cost | Implementation Plans to  upscale/imp element? | Maintenance Results sustained  (months)? | Maintenance When will  intervention  become  available? |
| --- | --- | --- | --- | --- | --- | --- | --- | --- | --- |
| Chang et al [38], 2022 | 72/88  82% | NS^c^ | Visiting breast surgery clinics, cancer wards, and breast cancer affiliate websites | 40/41(97.5%) | 2/41(0.05) | NS | NS | NS | NS |
| Compen et al [39], 2018 | 245/434  56% | Distress  NS | Health care professionals in six centers via online media, offline media, patient associations, and peers | 63/90(70%) | 18/90(0.20) | NS | NS | NS | NS |
| Kubo et al [40], 2018 | 772/1102 70.5% | NS | Clinic referrals from oncologists, oncology social workers, and nurses; brochures at each clinic; and invitation emails, followed up with phone calls | 40/54(70%), 65% practiced at least 50% of the days during the intervention period | 14/54(0.26) | 1 year app subscription | A year’s subscription to the mindfulness program used for this trial | NS | Approximately 70% of patients continued to use the program after the completion of the study |
| Kubo et al [37], 2019 | 103/796 13% | QoL^d^ 0.53 | Oncology clinicians; brochures at each clinic; and invitation emails, followed up with phone calls | 31/52(59.6%), 37 patients used the program for 50% - 70% of the time | 21/52(0.40) | 1 year app subscription | A year’s subscription to the mindfulness program used for this trial | NS | Approximately 96% of patients continued to use the program at least once after completion of the study |
| Liu et al [41], 2022 | 122/332 36.74% | Distress  –0.45(−3.15 to −0.39) | Researcher Hospital | 40/61(65.5), the home practice time in the intervention group ranged from 10 to 32 days | 21/61(0.34) | NS | NS | Results sustained  3-month post intervention | NS |
| Messer et al [42], 2020 | 23/NS | Anxiety and depression | Researcher Hospital | 11/11 (100%), exercises ranged from 8 to 17 min in length, with an average duration of 12 min | 1/23(0.48) | NS | NS | NS | NS |
| Nissen et al [34], 2018 | 150/389 39% | Anxiety or depression 0.45(0.26 to ‐0.64) | Researcher Hospital | 74/104 (71%), participants spent on average 6 hours and 38 minutes on each completed module, which would correspond to 2 hours for the session and 39 minutes of practice per day | 30/104(0.28) | The secure and encrypted intervention platform | Website with login provided access to daily exercises to participants | Results sustained  6-month post intervention | NS |
| Peng et al [33], 2022 | 60/65 92% | NS | Researcher Hospital | 28/30(93.3%) | 2/30(0.06) | NS | NS | Results sustained  1-month post intervention | NS |
| Rosen [35], 2017 | 95/116 82% | QOL  0.65 | Web-based data collection program | 31/48(64.5%) | 17/48(0.35) | 6-month app subscription | Headspace app publicly available | Results sustained  1-month post intervention | Headspace app publicly available |
| Rosen et al [36], 2018 | 112/137  82% | QOL  0.26 (1.69 to 10.55) | Researchers Flyers, letters, social  media, online message | 34/57 (59.6%), downloaded app  using activation code & used app  on mean 18/84 (21.4%) days | 18/57 (0.31) | 6-month  app subscription | Headspace app publicly available | Results sustained  1-month post intervention | Headspace app publicly available |
| Russell et al [43], 2019 | 69/120 58% | NS | Researcher Hospital | 32/46(72%) found the program helpful; 8 were not sure if the program was helpful | 14/46(0.30) | NS | NS | NS | NS |
| Shen et al [31], 202 | 77/NS | NS | Researcher Hospital | 37/40(92.5%), practice at least 6 MBCR courses, at least 6 days a week and at least 15 minutes a day | 3/37(0.08) | NS | NS | NS | NS |
| Wang et al [44], 2022 | 103/154 67% | NS | Nurses and researcher  Posters Hospital | 48/51(94.1%),the mean number of attended MBCR^e^ courses was 3.6 (SD 0.7; adherence rate=3.6/4, 90%) | 3/51(0.06) | A Chinese-version MBCR book and some assisted–mindfulness practice audios | NS | The effect was maintained at the 1-month follow-up | NS |
| Yousefi et al [32], 2022 | 50/87 57% | NS | Oncologist  Hospital | 23/25(92%), participants (experimental) logged internet for 9 weekly, 90 minutes online MBCR sessions | 2/25(0.08) | Internet access, recordings, manuals, and work books | NS | The effect was maintained at the 2-month follow-up | NS |
| Zernicke et al [45], 2014 | 62/67 93% | Feasibility  NS | Media outreach, promotional pamphlets, community-based networks, and mailing of study invitation letters | 25/30(83.3%), 30 immediate MBCR participants, 25 completed at least five or more classes (more than half the sessions) | 2/30(0.07) | Headsets, webcameras and MBCR program manuals and technical support | NS | NS | NS |

^a^RE-AIM: Reach, Effectiveness, Adoption, Implementation, and Maintenance

^b^95% Confidence Interval

^c^NS :not specified in the study

^d^QoL: quality of life

^e^MBCR: Mindfulness-based cancer recovery
